# Multiscale interface engineering in biohybrid composites for biomedical applications

**DOI:** 10.1016/j.mtbio.2025.102382

**Published:** 2025-10-04

**Authors:** Yi Wang, Tingyu Wang, Ran Tang, Dongtao Wang, Xianglong Zhang, Hiromi Nagaumi, Bowen Yang, Xu Ren, Jin Huang, Yingjie Zhang, Jiming Hao, Qiang Ma

**Affiliations:** a Sichuan Provincial Engineering Research Center of City Solid Waste Energy and Building Materials Conversion & Utilization Technology, Chengdu University, Chengdu, 610106, China; b High-Performance Metal Structural Materials Research Institute, Soochow University, Suzhou, Jiangsu, 215021, China; c School of Environment, State Key Joint Laboratory of Environment Simulation and Pollution Control, Tsinghua University, Beijing, 100084, China; d College of Agriculture and Biological Science, Dali University, Dali, 671000, China

**Keywords:** Multiscale composites, Interface engineering, Hierarchical design, Stimuli-responsive systems, Living biomaterials

## Abstract

Multiscale composites with engineered interfaces have emerged as a cornerstone in the development of next-generation biomedical materials. This review provides a comprehensive and structured overview of interface design strategies spanning nano-to macro-scales, emphasizing their role in modulating mechanical performance, biological signaling, and adaptive functionality. We categorize key approaches into three synergistic domains: hierarchical structuring for mechanical and cellular control, stimuli-responsive interfaces for dynamic biomedical functions, and bioinspired or living systems that mimic and integrate with biological environments. Advanced fabrication techniques—including additive manufacturing, surface nanofunctionalization, and layer-by-layer assembly—are reviewed alongside multiscale characterization tools for structural and interfacial analysis. We further link these interface strategies to a range of biomedical applications, such as osteochondral scaffolds, vascularized implants, antibacterial coatings, smart drug delivery carriers, and neural-integrated electronics. Biological interactions, including protein adsorption, mechanotransduction, and immune modulation, are explored to elucidate how engineered interfaces influence cellular fate and integration. Finally, we outline key challenges—such as manufacturing scalability, long-term biocompatibility, and regulatory approval—and propose forward-looking solutions enabled by AI-driven materials design and organ-on-chip validation. This review serves as a conceptual and technical roadmap for researchers developing multifunctional biomaterials through the lens of multiscale interface engineering.

## Graphical abstract

**Figure:**
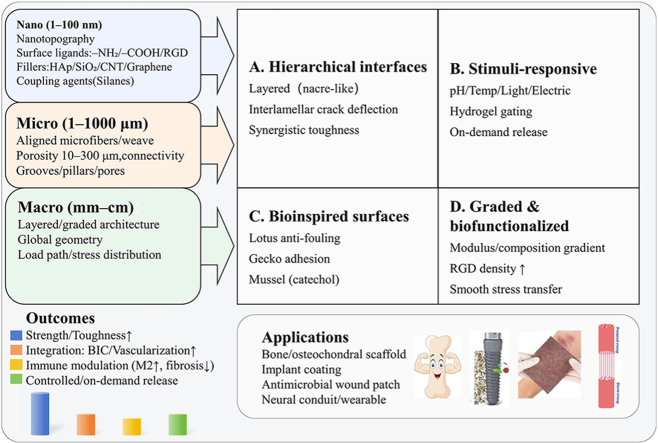

## 1 Introduction

Multiscale composite biomaterials—defined by structural integration from the nanometer to the centimeter scale—represent a paradigm shift in biomedical material science [1]. Unlike conventional monolithic materials, these composites allow the decoupling and tailored optimization of bulk mechanical properties, surface bioactivity, and degradation kinetics [2]. Central to this capability is **multiscale interface engineering**, which enables targeted control over interactions at each structural boundary—whether between distinct material phases or between the material and the host biological environment [3].

In biomedical applications, **interface zones are not passive junctions** but dynamic regulators of biological function. The surface is the first point of contact between a material and the body, and the biochemical and physical properties of this interface strongly dictate cellular responses including adhesion, proliferation, differentiation, and immune modulation [4]. However, traditional biomaterial design has often focused narrowly on **bulk properties**, with interface considerations limited to superficial modifications or simplistic coatings [5]. This reductionist approach neglects the complexity of biological systems, which themselves are organized hierarchically and respond to multi-scale cues [6].

**Multiscale interface engineering seeks to remedy this by designing interfaces that are spatially resolved, temporally dynamic, and biologically instructive** [7]. For instance, nanoscale features can guide protein adsorption and integrin clustering, microscale patterns influence cytoskeletal organization and tissue alignment, while macroscale gradients can mimic natural interfaces such as the osteochondral junction [8]. Such integration mirrors the architecture of native tissues, where function emerges from the orchestrated interaction of features across length scales [9].

Despite the conceptual appeal, significant challenges persist. Many existing studies demonstrate impressive short-term results under in vitro conditions but fail to translate into **stable, functional integration in vivo**. This disconnect often arises from inadequate consideration of the **dynamic biological context**, such as inflammation, remodeling, or aging. Moreover, **claims of “biomimicry” are often superficial**, replicating only the geometric features of natural tissues without emulating their spatiotemporal regulation or mechanical feedback loops. There is a pressing need for more rigorous frameworks to assess how interface design translates to biological outcomes—beyond static cell culture metrics [10].

This review argues that **true progress in biomedical composite materials demands a reconceptualization of interface design**: from surface decoration to multiscale, multifunctional engineering. We explore how hierarchical structuring, stimuli-responsive elements, and biomolecular functionalization can be integrated into coherent interface design strategies. We also highlight overlooked opportunities—such as the role of mechanotransduction pathways, immune interface tuning, and the incorporation of living or biohybrid components.

Finally, we emphasize that interface engineering must be understood not merely as a technical problem of surface modification, but as a **systems-level design challenge** bridging materials science, cell biology, and clinical need [11]. By critically reviewing current approaches, identifying limitations, and proposing future directions, this article aims to establish **multiscale interface design as a foundational paradigm** for the next generation of regenerative medicine, therapeutic delivery, and implantable devices.

This review's contribution. We contribute three concrete advances. First, we organize multiscale interface engineering across the nano–micro–macro spectrum into four design archetypes: hierarchical structuring, functionally graded interfaces, biofunctionalization, and stimuli-responsive/living interfaces. Second, we link each archetype to biological mechanisms and measurable outcomes through a concise strategy–mechanism–outcome map (e.g., bone–implant contact, fracture toughness/fatigue life, macrophage M2/M1 balance, release precision), so design choices are auditable and comparable. Third, we propose a translation checklist focused on manufacturability, long-term stability, and regulatory readiness, intended to streamline movement from bench to clinic. Together, these elements provide a practical framework for selecting, building, and validating multiscale interfaces for biomedical composites.

## 2 Core strategies for multiscale interface

**Fig. 1:**
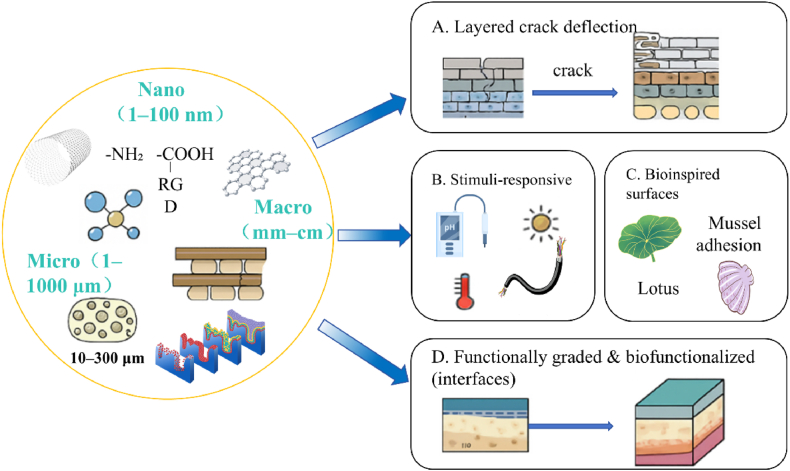
Core strategies and action pathways of multiscale interface engineering.

Multiscale interface engineering lies at the heart of advanced biomedical composite development [12]. Interfaces designed across multiple length scales—nano, micro, and macro—not only integrate diverse material properties but also modulate cellular and biochemical responses crucial for tissue integration and device performance [13]. This section outlines the major strategies employed in engineering such hierarchical interfaces, covering structural, responsive, and biological paradigms that collectively define the next generation of biofunctional materials, as in Fig. 1. Building on the scope and rationale set in the Introduction, this section distills the core interface strategies across the nano–micro–macro scales and how they work together.

Table 1 provides a structured overview of the key length scales involved in multiscale interface engineering, linking specific interface features to their functional significance in biomedical composite design. By highlighting representative examples at macro-, micro-, and nano-levels—including hierarchical and bioinspired systems—the table emphasizes the necessity of integrated, cross-scale strategies to achieve high performance in implantable and regenerative biomaterials.

**Table 1:** Key length scales in multiscale interface engineering and their roles.

Length Scale	Interface Features/Engineering Focus	Significance in Biomedical Composites	Ref.
Macroscale (mm)	Bulk architecture and structural design (e.g. layered or graded structures)	Provides structural integrity and load-bearing capacity for implants. Macro-scale design ensures the composite can support physiological loads and maintain shape.	14
Microscale (μm)	Micro-structural arrangement of fillers (fiber orientation, porosity)	Controls distribution of stresses and prevents stress concentrations. Proper microscale interface design improves dispersion of reinforcements and reduces defects.	15
Nanoscale (nm)	Surface chemistry and molecular bonding at interfaces	Nanoscale interactions (e.g. coupling agents, chemical bonds) ensure strong adhesion between phases. Influences overall composite properties via interfacial forces and helps transmit load across phases.	16
Hierarchical Multi-scale	Integrated design across nano-, micro-, and macro-scales	Combining features at multiple scales yields synergistic improvements in performance. Hierarchical interfaces can deflect cracks and dissipate energy across scales, enhancing toughness and reliability.	17
Natural Paradigms	Bioinspired multiscale interface architectures from nature	Nature provides blueprints of effective multiscale composites. Biological materials have multiple interface levels that optimize mechanical and biological function. Learning from these informs the scope of interface engineering.	18

### 2.1 Hierarchical structuring

#### 2.1.1 Mechanical reinforcement via multiscale interfaces (nano-micro-macro synergy)

Hierarchical interphases increase toughness and fatigue life by combining nanoscale bonding, microscale stress redistribution, and macroscale load paths [19]. Nano-scale fillers enhance interfacial bonding through high surface area and surface energy, micro-scale structures distribute stress uniformly, and macro-scale geometries provide bulk mechanical integrity [20]. This synergy leads to improved fracture toughness, fatigue resistance, and load-bearing capacity. Examples include nanoparticle-reinforced polymer matrices, micro-fiber embedded hydrogels, and graded composite scaffolds where each tier contributes to a distinct mechanical function [21].

#### 2.1.2 Controlled Degradation and structural integrity (graded porosity, sequential drug release)

Multiscale porosity design enables the spatial and temporal control of degradation and therapeutic release [22]. Nanopores can dictate molecular diffusion, micropores support cell infiltration, and macropores enhance vascularization and nutrient flow [23]. This hierarchical porosity is often coupled with bioresorbable polymers or ceramics that degrade at different rates, allowing sequential resorption or staged drug release. Such constructs can match the healing timeline of tissues, offering improved regeneration outcomes [24].

#### 2.1.3 Cellular responses to multiscale topography (adhesion, alignment, differentiation)

Cells interact with their microenvironment through mechanosensing machinery that detects features as small as a few nanometers. Hierarchical topographies mimic the extracellular matrix by integrating microgrooves, nanoridges, and surface roughness gradients [25]. These features guide cell adhesion, direct cytoskeletal organization, and influence lineage-specific differentiation—e.g., nanogratings promoting neural alignment or micropatterns enhancing myogenic differentiation. The ability to spatially orchestrate cell fate through designed topography is a hallmark of multiscale interface design [26].

### 2.2 Stimuli-responsive and adaptive interfaces -trigger classes and design windows

endogenous vs exogenous triggers. We categorize triggers as endogenous (host-derived: pH, enzymes, temperature, physiological strain) and exogenous (applied fields: light/NIR, electrical, magnetic, ultrasound). In this section, pH/temperature/enzymes/strain are treated as endogenous cues, while light/electric/magnetic/ultrasound are exogenous inputs that modulate interfacial properties.

#### 2.2.1 On-demand drug delivery 1 (pH/temperature-responsive polymers)

Endogenous pH and temperature cues are widely used to gate interfacial permeability and release at the material surface. Weak polyelectrolytes (e.g., poly(acrylic acid), polymethacrylic acid, chitosan derivatives) and acid-labile linkers (e.g., hydrazone, acetal) open or cleave in mildly acidic niches—tumor/wound pH ∼6.2–6.8 or endosomal pH ∼5–6—while remaining closed at physiological pH 7.4, enabling on-demand delivery. Thermo-responsive hydrogels (e.g., PNIPAm, LCST ∼32–37 °C; Pluronic-type block copolymers; elastin-like polypeptides) switch swelling/modulus near body temperature to modulate diffusion and release. Common architectures include core–shell nanoparticles, interpenetrating polymer networks (IPNs), and layer-by-layer (LbL) polyelectrolyte multilayers coated on porous scaffolds to stage early vs. late release. Representative set-points: pH gates 6.5–6.8 for opening; LCST near physiological for reversible thermal switching. Safety note: designs cap local ΔT ≤ 2–3 °C and constrain cumulative dose within tissue tolerance; examples and performance metrics are detailed in Section 4.3 (Smart Drug Delivery Systems).

#### 2.2.2 Dynamic implant interfaces (mechanical/thermal actuation, self-lubrication)

Dynamic interfaces adjust their properties in vivo to match the physiological environment. Self-lubricating surfaces reduce wear and inflammation in orthopedic implants, while thermoresponsive polymers modulate stiffness in response to body temperature, enhancing tissue compliance [27]. Mechanically actuated interfaces have been explored in cardiovascular stents and neural probes that require deployment or positional adjustments post-implantation. Actuation amplitudes are kept within physiological strain (≤1–2 % surface strain, 0.5–2 Hz) and surface temperature ≤39 °C to avoid peri-implant damage; shear-triggered lubrication typically engages around 10–100 s^−1^.

#### 2.2.3 Electro-biochemical responsiveness (neural interfaces, biosensors)

This subsection focuses on exogenous inputs—light (NIR), electrical and magnetic fields, and ultrasound—that gate adhesion, charge transfer, or permeability at the interface; pH- and temperature-responsive designs are treated under endogenous triggers (Sections 2.2.1–2.2.2) [28]. These systems are central to biosensors, neural electrodes, and electrically modulated drug delivery. Electro-responsive hydrogels, for instance, change volume upon voltage application, enabling electrically triggered molecule release or tissue stimulation [29]. Electrical stimulation is applied within 0.1–1 V mm^−1^ (or ≤0.35 mC cm^−2^ per phase, electrode-dependent) and 1–100 Hz, respecting tissue-specific safety limits.

### 2.3 Bioinspired and living interfaces

#### 2.3.1 Nature-inspired designs (gecko-adhesion, antifouling surfaces)

Bioinspiration offers rich templates for interface design. Gecko-inspired dry adhesives replicate hierarchical setae structures for reversible attachment in wet environments [30]. Antifouling surfaces mimicking shark skin or lotus leaves prevent microbial colonization and protein fouling through surface energy modulation and nanoscale roughness [31]. These biomimetic approaches translate evolutionary efficiency into functional biomedical interfaces.

#### 2.3.2 Living composites (cell-embedded matrices, bioactive components)

Living composites incorporate viable cells or biologically derived factors directly into the material [32]. These systems offer self-healing capabilities, dynamic remodeling, and biologically tuned responses [33]. Examples include stem cell-laden hydrogels for regenerative scaffolds and extracellular matrix–based coatings that actively recruit host cells [34]. Integrating living components necessitates precise control of microenvironments, including oxygen diffusion, mechanical support, and immunological shielding [35,36].

#### 2.3.3 Challenges in viability and functionality (immunoisolation, vascularization)

Embedding living systems into composites presents significant engineering challenges. Long-term viability requires nutrient delivery and waste removal, often demanding vascularization strategies or perfusable constructs. Immunoisolation barriers (e.g., hydrogel capsules, polymer membranes) are essential to prevent rejection in allogeneic or xenogeneic cell therapies. Ensuring functional integration between host and material necessitates balancing immune modulation, structural integrity, and dynamic adaptability [37].

Table 2 summarizes representative interface engineering strategies employed in multiscale biohybrid composites, highlighting their underlying mechanisms and functional advantages. From hierarchical architectures to bioinspired and stimuli-responsive systems, each approach contributes distinct mechanical, biological, or adaptive benefits. This comparative overview helps contextualize how different interface designs can be selected or combined based on application-specific requirements.

**Table 2:** Core interface design strategies in biomedical composites.

Design Strategy	Interfaces gated by endogenous cues (pH, enzymes, temperature, physiological strain) and/or exogenous inputs (light/NIR, electrical, magnetic, ultrasound) to switch adhesion, permeability, or release.	Advantages/Examples	Ref.
Hierarchical Interfaces	Building structures with multiple levels of organization (nano → micro → macro). Often inspired by biological composites, this involves creating an interfacial architecture at different scales (e.g. nano-coatings on fibers plus micro-scale fiber alignment).	Enhances mechanical synergy and toughness by crack deflection at interfaces across scales. Example: Nacre-like layered composites and bone-mimicking scaffolds use hierarchical interphases to achieve high strength and fracture resistance.	38
Stimuli-Responsive Interfaces	Incorporating materials that change properties in response to external stimuli (pH, temperature, light, stress, etc.). For instance, smart polymer coatings can swell, stiffen or expose/tether molecules upon stimulation.	Allows on-demand or environment-triggered changes in the interface, enabling controlled drug release or cell adhesion tuning. Such “smart” interfaces can alter structure or chemistry when triggered, providing in situ adaptability (e.g. a thermo-responsive hydrogel coating that releases growth factors when heated).	39
Bioinspired Surface Engineering	Designing interfaces inspired by natural surfaces and chemistry. Examples include superhydrophobic lotus-leaf‐like surfaces to repel fluids/bacteria, gecko-inspired nanopatterns for strong yet reversible adhesion, or mussel-inspired polydopamine coatings for underwater adhesion.	Yields special interfacial functions: e.g. Lotus-effect rough coatings give anti-fouling, self-cleaning properties by repelling proteins and microbes; Gecko/mussel-inspired adhesives provide strong wet adhesion to tissues. These strategies improve biocompatibility (reduced fouling) and fixation without toxic glues.	40
Functionally Graded Interfaces	Creating gradual transitions in composition or properties across an interface region, rather than a sharp boundary. For example, a implant coating that gradually changes from a hard, inorganic outer layer to a softer, polymer inner layer.	Minimizes stress discontinuities and mimics natural tissue transitions. Graded interfaces improve mechanical integration and reduce delamination. Example: Graded bone–tendon scaffold with a continuous mineral-to-collagen transition improves load transfer and tissue integration, unlike abrupt hard/soft junctions.	41
Biofunctionalization (Biochemical)	Modifying the interface with biological moieties (peptides, proteins, growth factors, etc.) or bioactive polymers. These coatings or grafted molecules specifically interact with cells or signaling pathways.	Promotes desired cellular responses and integration. For instance, coating a scaffold with RGD peptides or bone morphogenetic protein creates an interface that actively encourages cell attachment and differentiation. Such biofunctional interfaces can expedite tissue bonding and healing compared to inert surfaces.	42

Note: We use “endogenous” for host-derived cues (pH, enzymes, temperature/strain) and “exogenous” for applied fields (light/electric/magnetic/ultrasound). Earlier wording “external stimuli” has been clarified accordingly.

In sum, multiscale interface engineering provides a versatile toolkit to harmonize structural mechanics, biological compatibility, and dynamic responsiveness in biomedical composites. The following sections will elaborate on fabrication methods, characterization tools, and clinical application domains that leverage these core strategies. To avoid repetition, Section 2 presents design principles only; all application examples are consolidated in Section 4. These principles set up how such interfaces are built and verified, which we cover next in Section 3.

## 3 Advanced fabrication and characterization techniques

**Fig. 2:**
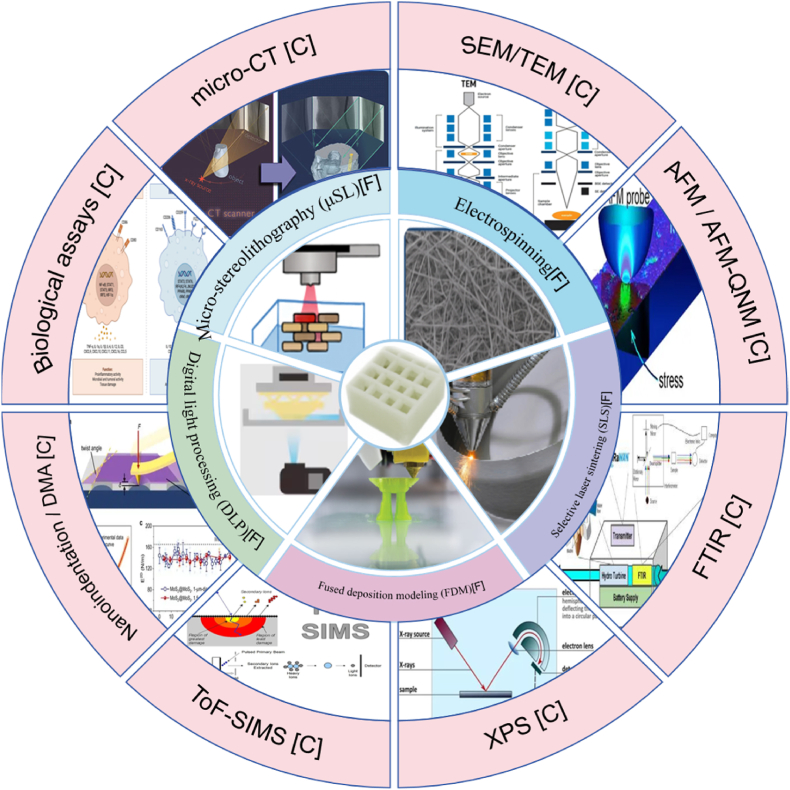
Fabrication and characterization techniques for multiscale interfaces. Fabrication = [F]; Characterization = [C].

The realization of functional multiscale interfaces relies heavily on advanced fabrication and characterization technologies capable of constructing and analyzing intricate hierarchical architectures [43]. From nano-structuring surfaces to embedding living cells within scaffolds, the convergence of engineering precision and biological relevance is key [44]. This section explores state-of-the-art techniques for both fabrication and evaluation of multiscale interface systems, as shown in Fig. 2 [45]. We now move from what to build to how to build and measure it, mapping each method to the interface features it enables and to readouts tied to biological endpoints.

### 3.1 Cutting-Edge fabrication

#### 3.1.1 3D bioprinting and additive manufacturing (hierarchical scaffolds, cell-laden bioinks)

3D bioprinting enables the layer-by-layer deposition of materials and cells with micrometer resolution [46]. Multimaterial printing allows the construction of scaffolds with spatially varying stiffness, porosity, and biochemical composition, mimicking the gradient complexity of native tissues [47]. Cell-laden bioinks composed of hydrogels, decellularized ECM, and growth factors support high cell viability and allow direct incorporation of biological functionality during the fabrication process [48].

#### 3.1.2 Nanofabrication & surface functionalization (lithography, plasma modification, LbL assembly)

Nanofabrication techniques such as electron-beam lithography, nanoimprinting, and soft lithography are instrumental in creating nanoscale features that guide cell behavior [49]. Surface treatments including plasma modification, silanization, and layer-by-layer (LbL) assembly provide precise control over surface energy, chemistry, and biofunctional ligand presentation [50]. These methods are essential for replicating the native extracellular matrix landscape at the nanoscale.

### 3.2 Multiscale characterization

We report interface readouts by domain—structure, chemistry, mechanics, biology—so that measurements align with the design levers in Section 2 and the application outcomes in Section 4.

#### 3.2.1 Structural analysis (Micro-CT, SEM/TEM, AFM)

Characterizing hierarchical architectures requires techniques spanning multiple resolution scales. Micro-computed tomography (micro-CT) offers 3D visualization of internal scaffold porosity and geometry [51]. Scanning and transmission electron microscopy (SEM/TEM) reveal fine structural details at the micro- and nanoscale [52]. Atomic force microscopy (AFM) not only provides topographic mapping but also quantifies local mechanical properties, such as stiffness gradients across interfaces [53].

#### 3.2.2 Interfacial property mapping (nanoindentation, FTIR/XPS spectroscopy)

Understanding interface behavior necessitates precise measurement of chemical and mechanical gradients [54]. Nanoindentation enables localized stiffness and hardness mapping, crucial for designing interfaces with mechanical matching to native tissues [55]. Spectroscopic techniques such as Fourier-transform infrared spectroscopy (FTIR) and X-ray photoelectron spectroscopy (XPS) are used to assess chemical composition, bonding states, and functional group distribution at composite interfaces [56]. These analytical tools are fundamental for validating the efficacy of functional coatings, ligand conjugation, and material modifications.

Together, these fabrication and characterization approaches provide the technical foundation for constructing and evaluating next-generation biomedical composites with tailored multiscale interfaces. In the next section, we will explore how these engineered systems are applied in practical biomedical contexts, ranging from tissue regeneration to bioelectronic integration.

Table 3 summarizes key fabrication and characterization techniques employed in interface-engineered biomedical composites. The listed methods span from structural fabrication (electrospinning, 3D printing, chemical functionalization) to advanced characterization (microscopy, spectroscopy, mechanical and biological testing), each contributing to interface construction, verification, and performance evaluation. The table illustrates how combining these methods allows precise design and validation of functional multiscale interfaces essential for clinical translation. With the toolchain in place, we turn to where these interfaces pay off—concrete biomedical applications in Section 4.

**Table 3:** Key fabrication techniques and characterization methods for interface-engineered composites.

Method (Type)	Description/Purpose	Application/Example	Ref.
Electrospinning (Fabrication)	Uses electrostatic forces to produce ultrafine fibers (nano- to micron-scale) from polymer solutions. Yields high-surface-area meshes that mimic ECM-like nanofibers.	Creates nano-fibrous scaffolds with tunable porosity and fiber alignment. Example: electrospun interfacial mats on implants improve cell attachment by simulating collagen fibers; often combined with 3D printing for mechanical support.	[57]
3D Printing/Bioprinting (Fabrication)	Additive manufacturing to build custom 3D structures layer-by-layer; precise control of macro-architecture and internal geometry (including gradients).	Enables patient-specific implants and multi-material interfaces. Example: bioprinted scaffolds with defined pore networks integrated with electrospun nanofibers form hierarchical, mechanically stable, cell-permissive constructs.	[58]
Surface Chemical Functionalization (Fabrication)	Silanization, plasma, or polydopamine coatings to modify filler/substrate surfaces; introduces chemical groups for interfacial bonding.	Improves compatibility between dissimilar phases. Example: silane coupling on glass/ceramic fillers forms covalent bonds with resin, strengthening the interphase and durability in dental/orthopedic composites.	[59]
Layer-by-Layer Assembly (Fabrication)	Sequential deposition of alternating polymer/nanoparticle/biomolecule layers; nanometer-scale thickness control.	Creates ultra-thin, controlled interfacial coatings with biofunction or protection. Example: LbL films immobilize growth factors at implant interfaces for sustained local bioactivity.	[60]
Scanning Electron/Transmission Microscopy (Characterization)	High-resolution imaging to examine surface topography, interphase morphology, and phase dispersion. SEM provides micro- to nanoscale visualization of composite cross-sections and interphase quality; TEM resolves nanostructures at the interface.	Verifies interface structure and uniformity. Example: SEM shows nanofiber distribution within pores; EDS maps elemental gradients (e.g., calcium at polymer–ceramic boundaries).	[61]
Spectroscopic Analysis (Characterization)	FTIR and XPS probe chemical bonds and surface composition at interfaces; FTIR identifies functional groups, XPS resolves elemental states.	Confirms successful surface modification/coupling. Example: FTIR peaks verify dopamine/oxidation chemistry; XPS detects bioactive ligands (e.g., nitrogen signal from RGD) on coated implants.	[62,63]
Mechanical Testing (Characterization)	Interfacial/mechanical performance via interfacial shear, fiber pull-out, nanoindentation, and DMA.	Ensures engineered interfaces meet mechanical requirements. Example: nanoindentation across fiber–matrix boundaries shows a modulus gradient (well-formed interphase); pull-out tests indicate higher debonding forces with optimized coupling; bulk tests show higher strength/toughness.	[64]
Biological Assays (Characterization)	In vitro/in vivo evaluation of cell–material interactions and immune responses (adhesion, proliferation, differentiation; cytokines/phenotypes).	Validates biocompatibility and functional performance. Example: higher osteogenic markers (ALP, OPN) on engineered interfaces; macrophage assays show M2 shift; animal studies report reduced fibrosis and faster integration.	[65]

## 4 Biomedical applications

**Fig. 3:**
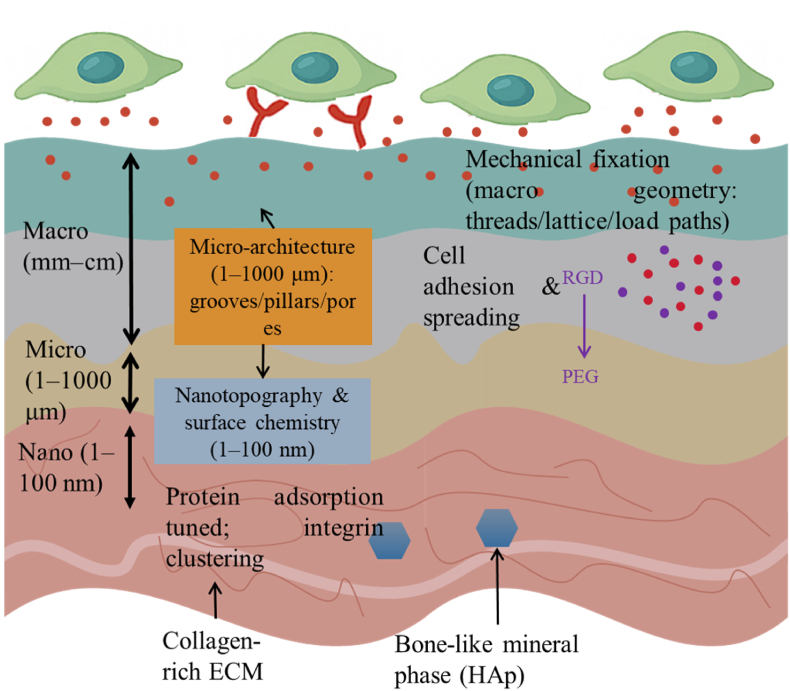
Multiscale surface features and their roles in interface performance.

Multiscale interface engineering has unlocked a new generation of biomedical composites tailored for specific clinical outcomes [66]. By integrating structural, chemical, and biological cues into functional interfaces, these materials support tissue regeneration, implant integration, targeted therapy, and biosensing [67]. This section highlights the main biomedical domains where such engineered interfaces are making impactful contributions. Fig. 3 links macro (mm–cm), micro (μm), and nano (nm) surface features to their dominant outcomes—mechanical fixation, tissue ingrowth/alignment, and protein-mediated cell adhesion/mechanotransduction, respectively. Each use-case is organized by the interface levers it employs and the measurable outcomes they drive, with pointers back to Sections 2, 3 where relevant.”

### 4.1 Tissue engineering scaffolds

#### 4.1.1 Osteochondral regeneration (stiffness-graded interfaces)

Functionally graded osteochondral scaffolds couple cartilage-like compliance with bone-level stiffness, preventing delamination and improving early fixation [68]. Multiscale interfaces allow for the fabrication of stratified composites—soft hydrogel-based regions promoting chondrogenesis and stiff ceramic-enriched regions supporting osteogenesis. Such constructs enhance integration with host tissue and promote spatially regulated cell differentiation, reducing the risk of delamination and failure [69].

#### 4.1.2 Vascularized constructs (dual-scale porosity, angiogenic cues)

Effective tissue regeneration demands rapid vascularization. Scaffolds with multiscale porosity—micropores for capillary ingrowth and macropores for perfusion—facilitate neovascularization [70]. Interfaces functionalized with angiogenic factors or adhesive peptides (e.g., VEGF, RGD) further enhance endothelial cell migration and tube formation, critical for long-term tissue viability and integration [71].

### 4.2 Implantable devices

#### 4.2.1 Osseointegration-enhanced coatings (nanotextured hydroxyapatite)

Orthopedic and dental implants benefit from surface modifications that promote bone anchoring [72]. Nanotextured hydroxyapatite coatings or titania nanotubes on titanium implants increase surface roughness and bioactivity, enhancing osteoblast adhesion and mineralization [73]. Hierarchical interfaces combining nanostructures with micro-porosity enable mechanical interlocking and biochemical signaling, significantly improving implant fixation and longevity.

#### 4.2.2 Antibacterial interfaces (biofilm-resistant nanotopographies)

Nanoscale bactericidal topographies reduce biofilm formation while preserving mammalian cell compatibility [74]. Interfaces can also be functionalized with silver nanoparticles, antibiotics, or antimicrobial peptides to provide sustained release, reducing biofilm formation and post-operative complications [75].

### 4.3 Smart Drug Delivery Systems

#### 4.3.1 Stimuli-responsive carriers (core-shell nanoparticles, IPN hydrogels)

Multiscale interfaces are central to responsive drug delivery platforms. Core-shell nanoparticles with pH- or redox-sensitive shells enable cargo release in targeted microenvironments such as tumors [76]. Interpenetrating polymer networks (IPNs) combine mechanical stability with responsiveness, enabling transdermal patches or injectable gels that respond to temperature or enzyme concentrations, allowing controlled and localized therapy [77].

#### 4.3.2 Multimodal release (photothermal-triggered therapeutics)

Combining multiple stimuli in a single interface allows greater control over drug kinetics [78]. Photothermal systems, such as gold nanorods or carbon nanostructures embedded in thermosensitive polymers, release drugs upon near-infrared irradiation. This dual-function interface enables simultaneous hyperthermia and drug delivery, improving cancer treatment efficacy while reducing systemic toxicity [79].

### 4.4 Biointegrated electronics

#### 4.4.1 Conformal wearable sensors (micropatterned polymer interfaces)

Wearable biosensors require flexible interfaces that conform to dynamic skin surfaces [80]. Micropatterned elastomeric films with conductive nanofillers (e.g., graphene, CNTs) provide both mechanical compliance and electrical conductivity. These materials enable real-time monitoring of physiological signals such as glucose, sweat electrolytes, or motion, with minimal irritation or detachment.

#### 4.4.2 Neural probes with living electrodes (cell-material hybrid interfaces)

Neural interfaces demand biocompatibility and signal fidelity. Hybrid electrodes that integrate living cells (e.g., astrocytes, neurons) with conductive polymers create biologically active interfaces that reduce gliosis and improve electrophysiological performance [81]. Such constructs maintain low impedance and facilitate seamless communication between electronic devices and neural tissue over extended periods [82].

Interface engineering plays a pivotal role in enhancing the performance and biological compatibility of biomaterials across a wide range of clinical contexts [83]. The table below (Table 4) highlights representative biomedical application domains—ranging from orthopedic and dental restoration to wound healing and cardiovascular interventions—along with the interface strategies employed and their corresponding outcomes. These examples illustrate how rational interface design can overcome traditional material limitations, facilitate tissue integration, and enable functional restoration.

**Table 4:** Selected biomedical applications and the role of interface engineering.

Application Area	Interface Engineering Strategy	Outcome/Advantage (Examples)	Ref.
Orthopedic (Bone Tissue Engineering)	Hierarchical, bioactive interfaces in composite bone scaffolds (e.g., collagen/nanoparticle coatings on porous scaffolds; mineralized nanofibers).	Enhanced osseointegration and mechanical performance. Example: a multi-scale scaffold with a 3D-printed framework plus electrospun nanofiber coating shows high initial strength and promotes osteoblast differentiation/mineralization; nano-HAp, RGD, etc., accelerate new bone formation and secure fixation.	84
Osteochondral (Bone–Cartilage Interface)	Functionally graded composite design—gradual transition from cartilage-like polymer to bone-like mineral; implemented via layered bioprinting or gradient fiber deposition.	Simultaneous regeneration of cartilage and subchondral bone with seamless integration. Example: a bioprinted osteochondral scaffold with stiffness/composition gradients supports chondrocytes on one side and osteoblasts on the other, mimicking the native transition and improving joint function.	85
	Silanized filler interfaces and bioactive coatings. In resin composites, organosilane coupling strengthens the resin–filler interphase; in implants, nanoscale calcium phosphate/peptide coatings on Ti.	Improved durability and bonding in the oral environment. Example: silane-engineered glass fillers tightly bond to resin (↑ strength/wear resistance); nano-HAp coatings on Ti promote early osseointegration vs uncoated surfaces.	
Dental Restoratives and Implants	Bioinspired adhesive/antibacterial interfaces in hydrogel dressings (e.g., nanofiber-reinforced hydrogels with catechol chemistry and/or antimicrobial agents).	Faster closure and better infection control. Example: a lotus-/mussel-inspired hydrogel (oxidized alginate/PAAm with cellulose reinforcement and dopamine chemistry) shows strong wet adhesion, self-healing, intrinsic antibacterial activity, and shortens full-thickness wound healing in mice.	86
Wound Healing and Skin Tissue	Stimuli-responsive composite carriers (core–shell nanoparticles; hydrogels with functionalized pore surfaces) designed to respond to pH/temperature or to target cell receptors.	Targeted, on-demand release with higher on-target exposure and lower variability. Example: pH-responsive nanocomposites dissolve an interface coating in acidic tumor microenvironments to release anticancer drugs precisely at the site; stimuli-sensitive hydrogels provide precise kinetic control.	87
Controlled Drug Delivery Systems	Hierarchical, bioactive interfaces in composite bone scaffolds (e.g., collagen/nanoparticle coatings on porous scaffolds; mineralized nanofibers).	Enhanced osseointegration and mechanical performance. Example: a multi-scale scaffold with a 3D-printed framework plus electrospun nanofiber coating shows high initial strength and promotes osteoblast differentiation/mineralization; nano-HAp, RGD, etc., accelerate new bone formation and secure fixation.	88

In summary, biomedical applications of multiscale interface engineering span a wide spectrum—from hard tissue regeneration to bioelectronic integration—each leveraging specific structural and functional capabilities. The strategic design of such interfaces enhances clinical performance, reduces complications, and opens new frontiers in personalized and regenerative medicine. The following section will focus on the biological mechanisms that underlie these material-tissue interactions. To unpack why these gains appear, Section 5 examines cell–material crosstalk and immune responses at engineered interfaces.

## 5 Interface-engineered composites vs conventional biomaterials: performance and applications

Why this comparison matters. Interface-engineered (IE) composites consistently outperform conventional materials on clinically relevant endpoints. Table 5 below summarizes typical ranges and why they matter in practice. Compared to conventional biomaterials, which are often limited by static mechanical and biological properties, interface-engineered composites exhibit tunable gradients, hierarchical structuring, and responsive functionalities that significantly enhance performance across a range of biomedical applications. The application results above are mediated by events at the interface; here we outline protein adsorption, adhesion/mechanotransduction, and innate immune polarization that govern integration.

**Table 5:** IE composites vs conventional biomaterials.

Aspect	Typical delta (IE vs no IE)	Clinical note
Early integration (BIC)	+20–40 %	Faster early fixation
Toughness/fatigue life	+30–100 % (or 2–5 × fatigue life)	Fewer load-related failures
Immune profile (M2/M1)	∼1.5–3 ×	Less fibrosis/inflammation
Release precision (CV)	−30–60 %	Lower dose, fewer off-target effects
Durability/wear/corrosion	Failure rate ↓; service life ↑	Fewer re-interventions

### 5.1 Performance advantages of interface-engineered composites

#### 5.1.1 Enhanced Tissue Integration

Enhanced Tissue Integration. One of the most compelling advantages is the improved integration with host tissues [89]. Traditional single-phase scaffolds, while offering structural support, often fail to recapitulate the complex zonal organization of native tissues, leading to poor regeneration outcomes [90]. In contrast, composite scaffolds with graded or multiphasic interfaces—particularly in osteochondral applications—better emulate the natural transition from cartilage to bone [91]. This structural biomimicry enhances tissue ingrowth, vascularization, and long-term functionality. Several studies have demonstrated that continuous compositional gradients improve nutrient transport and cellular adhesion more effectively than abrupt or homogeneous transitions [92].

#### 5.1.2 Improved Cellular Responses

Improved Cellular Responses. Multiscale topographical and chemical cues at engineered interfaces provide spatially controlled environments for cell attachment, proliferation, and lineage-specific differentiation [93]. Nanoscale surface roughness, micropatterning, and stiffness gradients have all been shown to influence stem cell fate decisions [94]. For instance, porosity-gradient scaffolds induce region-specific osteogenic differentiation, offering a level of biological control that uniform scaffolds cannot. These features collectively enable a biomaterial to interact with cells in a way that resembles the dynamic extracellular matrix (ECM), an ability that monolithic biomaterials fundamentally lack [95].

#### 5.1.3 Mechanical Reliability and Fatigue Resistance

Mechanical Reliability and Fatigue Resistance. Traditional implants often suffer from stress concentration, brittle failure, or delamination under cyclic loading [96]. Interface-engineered composites mitigate these issues by decoupling surface compliance from bulk stiffness. For example, nano-reinforced interfaces improve fracture toughness, while aligned microfibers distribute mechanical stress more evenly across the material. Bioinspired architectures—such as brick-and-mortar or nacre-like structures—demonstrate superior toughness and damage tolerance, particularly critical for load-bearing orthopedic or dental implants [97].

#### 5.1.4 Controlled Degradation and Drug Release

Controlled Degradation and Drug Release. Unlike uniform materials, interface-engineered systems can spatially and temporally tune degradation and drug delivery profiles. For example, a scaffold with a degradation gradient can release bioactive agents in a staged manner, matching the physiological healing process [98]. This has profound implications for regenerative medicine, as it allows for controlled support of different tissue formation phases. Similarly, smart wound dressings that incorporate pH- or light-responsive elements at their interfaces enable precise, on-demand drug release—a marked improvement over constant or passive drug-eluting systems.

#### 5.1.5 Biological Signaling and Immunomodulation

Biological Signaling and Immunomodulation. Interface-functionalized composites can present bioactive ligands or surface chemistries selectively, modulating host responses more precisely than conventional surfaces. For example, nanoscale coatings with osteoinductive peptides promote bone formation while minimizing fibrotic encapsulation [99]. By contrast, conventional metallic or polymeric surfaces often provoke foreign body reactions due to lack of bioactivity or improper topography [100]. The ability to fine-tune immunological interactions through interface design is particularly relevant for long-term implant survival and patient safety [101].

### 5.2 Translational case studies

Agili-C® Biphasic Osteochondral Implant. This FDA-cleared scaffold integrates distinct cartilage and bone phases to treat knee lesions [102]. In clinical trials, patients showed superior functional recovery and reduced need for joint replacement compared to microfracture surgery [103]. The interface-engineered design proved critical: mimicking the osteochondral transition allowed the scaffold to support both chondrogenic and osteogenic healing. This case exemplifies how material-phase continuity and biological zoning dramatically enhance therapeutic efficacy [104].

Hydroxyapatite-Coated Implants. Dental and orthopedic implants coated with hydroxyapatite (HA) exhibit significantly faster and stronger osseointegration compared to uncoated titanium [105]. Clinical data reveal enhanced early-stage bone-to-implant contact, translating to improved initial stability, especially in osteoporotic patients [106]. The HA layer acts as a biomimetic interface that enhances protein adsorption and bone-cell recruitment—functions absent in bare metal. Yet, challenges such as coating delamination or long-term resorption remain, highlighting the need for further innovation in interface bonding.

Heparin-Bonded Vascular Grafts. Surface-modified ePTFE grafts incorporating immobilized heparin have demonstrated reduced thrombogenicity and improved patency in vascular bypass procedures [107]. By engineering an antithrombotic interface, these grafts mimic the endothelial surface, delaying occlusion and reducing the frequency of reintervention. However, such chemical modifications must maintain long-term stability in blood-contact environments, a nontrivial engineering hurdle.

Triphasic Ligament-to-Bone Scaffolds. Preclinical studies employing tri-layered scaffolds for enthesis regeneration have shown the successful formation of region-specific tissue types. The ability to guide tissue morphogenesis along a spatial gradient of mineralization is a powerful validation of interface-engineering strategies. While promising, the reproducibility of these complex constructs and their immunogenicity in large animals or humans remain key barriers to translation.

Stimuli-Responsive Drug Delivery Patches. Recent wound dressings featuring composite hydrogels with embedded nanoparticles and responsive linkers demonstrate effective infection control through pH- or NIR-triggered antibiotic release [108]. These dynamic interfaces outperform static release systems by synchronizing therapeutic delivery with pathological triggers. Though technologically advanced, concerns around the cost, regulatory acceptance, and robustness under clinical conditions must be addressed before widespread deployment.

### 5.3 Critical reflections and design considerations

While interface-engineered composites clearly surpass traditional biomaterials in many metrics, their complexity introduces challenges [109]. Fabrication reproducibility, long-term reliability, and cost-effectiveness must be rigorously evaluated. Moreover, many reported benefits—such as enhanced differentiation or integration—derive from small-animal models or in vitro studies, necessitating further validation in clinical contexts [110].

Nonetheless, the convergence of biofabrication, nanotechnology, and synthetic biology is rapidly advancing this field. Future directions include the integration of AI-driven design tools, patient-specific material customization, and real-time biosensing feedback loops. Overall, interface engineering is not merely a material enhancement strategy—it is becoming the foundational principle of next-generation biomedical device design.

To further illustrate the value of interface engineering, Table 6 provides a direct comparison between interface-engineered composites and conventional biomaterials across key performance dimensions. The comparison highlights substantial improvements in mechanical properties, biological response, immunomodulation, and functional longevity, demonstrating how rational interface design elevates biomedical material performance far beyond traditional approaches. These mechanisms define what must be controlled and tested for translation; Section 6 summarizes challenges, a practical translation checklist, and future directions.

**Table 6:** Comparison of interface-engineered composites with conventional biomaterials.

Aspect	Interface-Engineered Composites	Conventional Biomaterials (No Interface Engineering)	Ref.
Mechanical Strength & Toughness	Strong interfacial coupling improves load transfer; hierarchical/graded interlayers act as crack arresters/bridges, raising toughness and fatigue life. Example: fiber composites with nano-engineered interphases show higher tensile strength vs the same systems without interface treatment.	Interfaces often act as weak links where cracks initiate; poor stress transfer and delamination lead to lower effective strength and brittle failure, especially in monolithic or uncoupled composites.	[111]
Biological Integration	Bioactive/biomimetic surfaces (e.g., bioceramic coatings, ligand-functionalized interfaces) promote direct tissue bonding, firm anchorage, and reduced fibrous encapsulation; “third-generation” designs provide instructive cues for regeneration.	Frequently bioinert and non-bonding; the body may wall off the implant with a fibrous capsule. Integration relies on slow ongrowth, risking gaps and inferior long-term fixation.	[112,113]
Immune Response	Surfaces can be tuned to be immunomodulatory—biasing macrophages toward M2 (pro-healing) over M1 (pro-inflammatory)—and can locally release anti-inflammatory agents, reducing chronic inflammation.	Untailored surfaces may sustain M1 dominance and foreign-body reactions, leading to fibrosis; often requires systemic management and can prolong healing or increase failure risk.	[114,115]
Multi-functionality	Interfaces can add functions (local drug delivery, antimicrobial action, sensing/monitoring, conductivity), addressing multiple clinical needs within one platform.	Typically single-purpose; extra functions require separate systems, reducing synergy (e.g., mechanical support without active infection control or guided healing).	[116]
Longevity & Durability	Robust matrix–filler coupling and barrier interlayers suppress debonding, wear particles, and corrosion; e.g., silane-engineered dental composites resist water ingress and filler leaching, extending service life.	Earlier interface degradation (water ingress, microcracks), faster wear/corrosion, and higher likelihood of replacement due to lack of interface optimization.	[117]

## 6 Biological interactions at engineered interfaces

Clinical translation hinges on controlling interface identity, process windows, and long-term stability under physiological loads. Interfaces are not passive boundaries; they actively mediate communication between synthetic materials and biological systems [118]. This chapter explores the cellular and immunological processes that occur at these interfaces and discusses strategies to harness or modulate them for better biomedical outcomes. Having linked design, tools, and biology, we now focus on what limits translation and how to retire those risks.

### 6.1 Cell-material crosstalk

#### 6.1.1 Protein adsorption and cell adhesion (surface chemistry/topography effects)

The initial biological response to any implanted material is protein adsorption [119]. The composition, conformation, and orientation of these adsorbed proteins are dictated by surface properties such as hydrophilicity, charge, and topography [120]. Nanoscale roughness enhances the adsorption of adhesive proteins like fibronectin and vitronectin, promoting integrin-mediated cell adhesion [121]. Chemical modifications using functional groups (e.g., –NH_2_, –COOH) further modulate cell behavior by affecting protein affinity and spatial distribution.

#### 6.1.2 Mechanotransduction-driven differentiation (substrate stiffness, nanotopographical cues)

Cells interpret mechanical signals from the substrate through focal adhesions and cytoskeletal tension, translating them into biochemical responses—a process termed mechanotransduction [122]. Substrate stiffness influences stem cell fate: softer matrices promote neurogenesis, while stiffer ones induce osteogenesis [123]. Multiscale topographies, such as ridges and pits, further modulate cytoskeletal alignment and nuclear morphology, affecting gene expression and differentiation.

### 6.2 Immune and integration responses

#### 6.2.1 Immunomodulatory interfaces (anti-fibrotic peptides, "self" markers)

The immune response to implanted materials determines long-term success [124]. Conventional biomaterials often trigger foreign body reactions, leading to fibrosis or encapsulation [125]. To mitigate this, engineered interfaces are functionalized with immunomodulatory agents such as anti-inflammatory peptides, CD47-mimicking ligands ("don't eat me" signals), or cytokine-sequestering domains. These strategies reduce macrophage activation, polarize immune cells toward pro-regenerative phenotypes, and enhance tissue integration.

#### 6.2.2 Strategies for neovascularization (growth factor-eluting interfaces)

Sustained vascularization is essential for the survival of implanted materials and the host response [126]. Interface designs that incorporate slow-release reservoirs of VEGF, bFGF, or other angiogenic factors promote endothelial recruitment and vessel formation [127]. Multiscale porous structures provide migration pathways for endothelial cells and support the deposition of extracellular matrix, synergizing with biochemical cues to accelerate neovascularization [128].

Collectively, these biological responses underscore the importance of interface design in determining material fate in vivo. Through the deliberate control of surface chemistry, mechanical cues, and bioactive presentation, multiscale interfaces can direct cell adhesion, guide lineage commitment, and orchestrate immune tolerance—paving the way for more reliable and integrated biomedical devices. The next chapter will focus on the practical challenges and translational outlook associated with these innovations.

## 7 Challenges and Future Perspectives

**Fig. 4:**
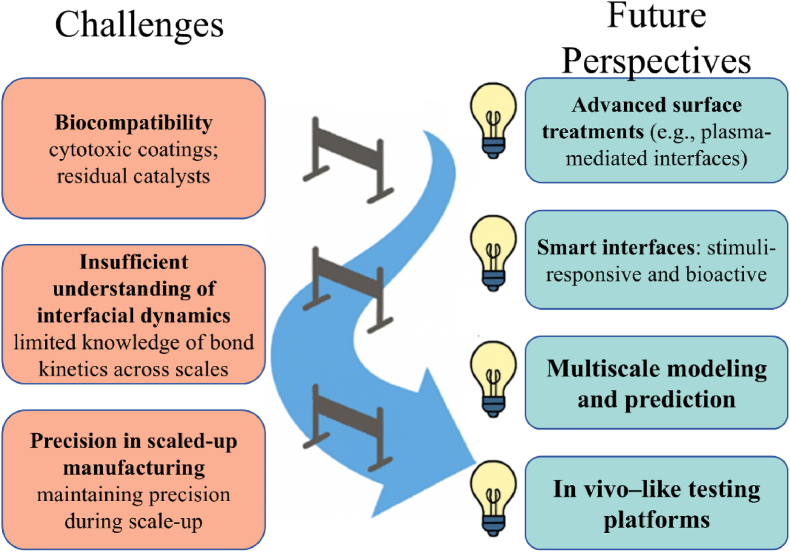
Challenges and future perspectives.

Despite the transformative potential of multiscale interface engineering in biomedical applications, several challenges remain that hinder clinical translation and widespread adoption. These include issues related to fabrication complexity, long-term biocompatibility, and regulatory pathways, alongside emerging opportunities through convergence with digital technologies and synthetic biology, as in Fig. 4.

### 7.1 Manufacturing complexity and scalability

Multiscale composites often require intricate fabrication processes that integrate multiple scales, materials, and functionalities in a single platform. Techniques such as lithography, layer-by-layer assembly, and multi-material 3D bioprinting are powerful but may be time-consuming, expensive, or incompatible with mass production. Ensuring reproducibility across batches and maintaining fidelity during upscaling are ongoing hurdles. Streamlining these manufacturing pipelines—through modular design, automated assembly, or hybrid fabrication systems—will be essential for industrial viability.

### 7.2 Long-term stability of living composites

Living interfaces, which incorporate cells or biological products, offer adaptive and regenerative functions but pose stability concerns. Viability of embedded cells may decline over time due to limited nutrient diffusion, immune reactions, or loss of phenotype. Engineering vascularized constructs or using encapsulation techniques to create immunoprotective microenvironments can improve longevity. Furthermore, synthetic biology may enable controllable gene circuits or feedback mechanisms to sustain functional output.

### 7.3 Biocompatibility and regulatory hurdles

The complexity of multiscale materials complicates safety evaluation. Nanoparticles or bioactive surfaces may interact unpredictably with tissues, leading to toxicity or inflammation. Dynamic, stimuli-responsive systems add another layer of regulatory uncertainty due to their changing nature. Standardization of testing protocols, development of predictive in vitro/in silico models, and early engagement with regulatory agencies will be critical for approval and clinical adoption.

### 7.4 Convergent innovations

The integration of interface engineering with artificial intelligence, systems biology, and organ-on-a-chip technologies holds promise for accelerating design and validation. AI algorithms can optimize material parameters based on biological data, while digital twins of tissue-material interactions may predict long-term performance. Organ-on-chip systems offer physiologically relevant platforms for preclinical testing, bridging the gap between bench and bedside.

### 7.5 Toward clinical translation

Future biomedical devices will likely feature active, intelligent interfaces capable of responding to patient-specific cues. Smart implants that self-adjust to mechanical or inflammatory signals, or self-healing materials that regenerate functional interfaces after damage, are on the horizon. However, successful translation will depend on multidisciplinary collaboration among engineers, biologists, clinicians, and regulatory experts to address integration, manufacturability, and long-term safety.

Practical translation checklist for interface-engineered composites.

• Material identity & traceability. Define interfacial chemistry (ligand type/density, coupling agents), particle specs (size/shape/surface), and any sterilization effects on the interface.
• Process windows & QC. Lock validated ranges for printing/coating/crosslinking; specify in-line/batch QC for interface metrics (e.g., coating thickness, roughness, ligand density).
• Batch-to-batch interface metrics. Track modulus gradient (ΔE) across the interphase, adhesion strength, thickness uniformity, and porosity/connectivity relevant to function.
• In vitro ↔ in vivo correlation. Predefine a small panel linking cell/immune readouts (ALP/OPN, M2/M1, cytokines) to animal outcomes (BIC%, fibrosis score), with acceptance thresholds.
• Failure modes & durability. Assess delamination, wear particle generation, corrosion, and function under cyclic load; include fatigue and accelerated aging where applicable.
• Regulatory path (predicate/combination). Clarify whether the product is device-only, drug–device, or living product; specify release specs and biocompatibility testing (e.g., ISO 10993).
• Shelf-life & sterilization stability. Define storage conditions and show that interface chemistry/performance remains within spec after real-time/accelerated aging and after the chosen sterilization method.

In summary, while multiscale interface engineering presents a compelling pathway toward next-generation biomedical solutions, realizing its full clinical potential requires addressing technical, biological, and regulatory barriers. At the same time, emerging synergies with AI, biofabrication, and synthetic biology offer exciting opportunities to transform these challenges into drivers of innovation.

## CRediT authorship contribution statement

**Yi Wang:** Project administration. **Tingyu Wang:** Writing – review & editing. **Ran Tang:** Writing – review & editing. **Dongtao Wang:** Funding acquisition. **Xianglong Zhang:** Methodology. **Hiromi Nagaumi:** Methodology. **Bowen Yang:** Resources, Project administration. **Xu Ren:** Resources. **Jin Huang:** Validation, Software. **Yingjie Zhang:** Software, Resources, Project administration. **Jiming Hao:** Validation, Supervision, Software. **Qiang Ma:** Writing – review & editing, Writing – original draft.

## Declaration of competing interest

The authors declare that they have no known competing financial interests or personal relationships that could have appeared to influence the work reported in this paper.

## Data Availability

Data will be made available on request.
